# Anatomical predicting factors of difficult spinal anesthesia in patients undergoing cesarean section: An observational study

**DOI:** 10.12669/pjms.35.6.1276

**Published:** 2019

**Authors:** Simin Atashkhoei, Saeed Samudi, Naghi Abedini, Nahid Khoshmaram, Masoumeh Minayi

**Affiliations:** 1 Simin Atashkhoei, Department of Anesthesiology, Al-Zahra Hospital, Tabriz University of Medical Sciences, Tabriz, Iran; 2 Saeed Samudi, Anesthesiologist, Shohada Hospital, Tabriz University of Medical Sciences, Tabriz, Iran; 3 Naghi Abedini, Assistant Professor of Anesthesiology, School of Medicine, Tabriz University of Medical Sciences, Tabriz, Iran; 4 Nahid Khoshmaram, Department of Medical Library and Information Sciences, School of Management and Medical Informatics, Tabriz University of Medical Sciences, Tabriz, Iran; 5 Masoumeh Minayi, Nursing Department, Shahid Madani Hospital, Tabriz, Iran

**Keywords:** Cesarean Section, Anatomical Factors, Spinal anesthesia

## Abstract

**Objectives:**

Although Spinal anesthesia is the most common and safe anesthetic method for patients undergoing cesarean section, difficult access to it is a frequent problem in operating theaters. The predictive factors for the difficulty of spinal anesthesia in patients undergoing cesarean section were investigated.

**Methods:**

A total of 110 pregnant women, single-stranded, aged 18-40 years old and ASA class I or II candidates for elective cesarean section with spinal anesthesia were studied. Demographic information, body appearance, ability to bend the back of the patient was recoded. Also the position of the anatomical landmarks of the lumbar spine, the presence or absence of deformity in the spinal column lumbar was recorded for all patients.

**Results:**

The correlation coefficient of age, weight, body mass index, general body appearance, retention ability, anatomical signs of the spinal column (touching the spinous process) and the interval between the vertebra with the difficulty of spinal anesthesia were statistically significant (*p*<0.05). Complications after spinal anesthesia had a statistically significant relationship with the difficulty of performing spinal blockade (*p*: 0.006).

**Conclusion:**

Increasing age, weight, body mass index, reducing the ability to bend the waist, the non-touching of the spinous process and interstitial space causes the difficulty of performing spinal anesthesia in patients undergoing cesarean section. The results can contribute to determining and designing a spinal blockade scoring system based on the patient’s characteristics and effective factors before the surgery, to facilitate the technique by anesthesiologist.

## INTRODUCTION

Spinal anesthesia is the most common and safe anesthetic method for patients with cesarean section.^1^,^2^ Among factors that affect the decision of anesthesiologist to perform spinal anesthesia severity of spinal anesthesia and the experience of anesthetist who carries out spinal anesthesia make sense.^3^ On the other hand difficulty in performing secure airway during pregnancy makes the regional anesthesia as a choice technique in these patients.^4^,^5^ Some studies have shown that anatomical signs behind the patient have a diagnostic value in predicting the ease of noraxial techniques.^6^,^7^ Previous studies showed that the anatomical signs of the back, visibility of vertebrae and the radiological features of the lumbar vertebrae are two independent factors predicting the spinal anesthesia outcome and the anesthetist’s experience was not a determining factor in the difficulty of performing the technique.^8^,^9^ Shankar et al. described the insufficient gap between the spinal vertebrae as the only problem for this technique.^10^

In many studies, the factors determining the ease of spinal anesthesia are described as age, sex, body mass index, deformity of the spinal vertebrae, anesthetist’s experience, the ability to identify land marks of spinal cord, spinal radiological features, the place of spinal anesthesia, skin distances from epidural and subarachnoid space, and the size and type of needles.^10^-^13^ In general, studies have shown that prenatal prediction of the ease of performing spinal anesthesia increases the chance of success, while preventing multiple punctures and increasing the level of comfort and quality of patient health.^14^,^15^ Considering that in previous studies, the best anatomical indications and grading of the ease for predicting the difficulty of the neuraxial techniques were not mentioned.^7^,^10^, the purpose of this study was to investigate the factors predicting the difficulty of performing spinal anesthesia in patients undergoing cesarean section.

## METHODS

This is a prospective observational study, in which the difficulty of spinal anesthesia (SA) technique was considered as the main variable. The results of a pilot study, with 10 samples, showed more than 70% of the ease of performing SA technique. Considering α = 0.05, the power of 80% and the difference of 15% in the difference in the SA difficulty, 103 samples were estimated. To increase the validity of the study, 110 samples were studied. After obtaining ethics committee approval from research deputy of Tabriz Universrity of Medical Sciences (No. 5/4/15020/04/12/26, 94/12/26) total of 110 pregnant women, single stranded, aged 18-40 years old and ASA class I or II, who were candidates for elective cesarean section with SA were studied. Exclusion criteria were gestational age less than 36 weeks, history of spinal surgery, allergy to anesthetic drugs, emergency cesarean section, systemic diseases, mental illness and fetal problems. Body habitus parameters were recorded as normal, lean and obese states. Abdominal flexor can be expressed as the maximum flexural strength in the standing position. Also the level of bending was recorded in three concave, straight, and convex. The straight or concave condition was considered as incapable in bending back. The patients were sitting in standard position for spinal anesthesia with head on the chest, curved lumbar and forward shoulders. The anatomical landmarks of the lumbar spine were evaluated on the basis of observation and touch of spinous process in three degrees: Grade I (characteristic of the carnation is quite evident); Grade II (the crop is not visible but is palpable) and Grade III (cropping is neither visible nor palpable). The presence or absence of deformity in the lumbar spine examined by the observation, touch of spinous process and any visible deviations from the midline (as the deformity of the lumbar vertebrae: scoliosis, kyphosis and lordosis) was taken as difficult SA. Interspace gap graded on touching the inter-vertebral space noted as good (The inter-vertebral space is easily touched), weak (hardly touched), and none (not palpable). SA performed by an anesthesiologist with experience of more than 20 years in sitting position with Quincke 25 in intervertebral space L4-5 or L3-4 with midline method by bupivacaine 0.5% (10mg) and fentanyl (20μg) in 2.2ml total volume. To assess the difficulty of performing a SA technique, the number of redirections, attempts in the same or another level of intervertebral space, needles used in the same size or larger, the use of paramedian approach and finally failure in performing the technique were scored at seven degrees (0-6) as following: Score 0: The first effort without needle movement, Score 1: The first attempt with one or two needle redirections, Score 2: The first attempt with more than two needle redirections, Score 3: New attempt in the same or another level of intervertebral space, Score 4: New attempt with paramedian approach, Score 5: New attempt with another needle in the same size or larger, Score 6: Failure in performing the technique. Ultimately, the ease or difficulty of performing SA was graded as follows: Score 0-1: easy, Score 2-3: moderate, Score 4-5: difficult, Score AND 6: impossible or failure, At the end, the complications of SA (headache, paresthesia and epidural hematoma), the duration of blockade, and the next anesthetic plan were recorded in the questionnaire.

The data were analyzed using descriptive statistical methods such as mean ± standard deviation, frequency (percentage) and mean difference test for independent groups and Chi square test for comparing qualitative variables between two groups or Fisher exact test using SPSS software version 15. A *P*-value less than 0.05 is considered as statistically significant.

## RESULTS

One patient was excluded from the study due to lack of adequate SA level for general anesthetic surgery and finally, 109 patients were analyzed. Of the nine patients suffering from complications of anesthesia, 6 (66.7%) had difficult SA, indicating a direct relationship between complications with the degree of SA difficulty. Demographic characteristics of patients are seen in Table-I. The preliminary analysis in the dispersion graphs showed a uniform relationship between the age and severity of SA, which was followed by a positive correlation between age and spinal anesthesia with a correlation coefficient of 0.226 (*P*: 0.019). The increase in age was associated with an increased probability of difficulty in the SA (OR: 1.153, CI 95%: 0.99-1.116 and *P*: 0.079). There was a positive correlation between weight and SA with a correlation coefficient of 0.29 (*P*: 0.002). Weight gain based on the logistic test was associated with a higher probability of difficulty in SA (OR: 1.51, CI 95%: 1.094-1.010 and *P*: 0.015). The correlation between height and SA had a coefficient (0.053) close to zero (*P*: 0.723). A decrease in height was associated with an increased probability of SA (OR: 0.047, CI 95%: 1.05884-0.884). This increase was not statistically significant. The correlation between BMI and SA had a correlation coefficient of 0.361 (*P* = 0.005). The increase in BMI was based on the logistic test, with an increased probability of difficulty in spinal anesthesia (OR: 1.159 CI 95%: 1.1246- 1.077), which was statistically significant. The probability that patients with a BMI of less than or equal to 30 had difficulty with SA than those with a BMI greater than 30 (OR: 1.128*, P*: 0.826). There was no significant relationship between the history of spinal anesthesia with SA (*P*: 0.352). The probability of SA was lower in patients with previous SA than in patients with no history of SA (OR: 0.91, CI 95%: 0.5444-0.449 and *P*: 0.327). The probability of SA was lower in housewives compared to employee (*P*: 0.072). Mean ± SD of sensory block duration was 31.04 ± 10.42 which was a significant positive correlation between duration of spinal anesthesia and SA (95% CI: 0.666 and *P*: 0.005). The general appearance of the body with SA hardness had a correlation coefficient of 0.211 (*P*: 0.013). The probability of SA was lower in patients with apparently thin appearance than those who were obese in appearance (OR: 0.13, CI 95%: 0.1-2.18), (*P*: 0.072).

**Table I:** Demographic characteristics of patients.

Variables	Mean ± SD
Age (years)	40.3±17.6
Weight (kg)	82.64±13.79
Height (cm)	160.22±6.21
BMI (kg/m^2^)	32.25±4.99
***Previous history of spinal anesthesia***	
No history of spinal anesthesia; n (%)	36 (33.02)
Positive history of spinal anesthesia; n(%)	73 (66.97)
***Occupation***	
Housewife; n (%)	73 (67%)
Employee; n (%)	36 (33%)
***Body appearance***	
Thin; n (%)	58 (53.21)
Obese; n (%)	45 (41.27)
***Lumbar spine deformity***	
Normal; n (%)	80 (73.4)
Scoliosis; n (%)	7 (6.4)
Kyphosis; n (%)	6 (5.5)
Lordosis; n (%)	16 (14.67)
***Ability to bend the waist***	
Straight; n (%)	76 (69.72)
Concave; n (%)	16 (14.67)
Convex; n (%)	17 (15.59)
***Anatomical signs***	
Visible and touchy; n (%)	20 (18.3)
Palpable; n (%)	69 (63.3)
Non; n (%)	19 (17.4)
***Ientervertebral interval***	
Palpable; n (%)	24 (22)
Hardly palpable; n (%)	73 (67)
Not palpable; n (%)	12 (11)

The probability of SA in patients with normal appearance was approximately equal to those with apparent obesity [OR: 1. 081, CI 95%: 2.546-0.459, *P*: 0.859]. Ability to bend the waist Out of 109 patients, 76 (69.72%) cases were straight, 17 (15.59%) were convex, and 16 (14.67%) were concave. The ability to bend the back with SA was poorly correlated with a correlation coefficient of 0.276 (*P*: 0.001). The probability of SA was lower in patients with convex waist than patients with concave lumbar spine (OR: 0.832, CI: 3.62-0.219). The probability of SA was higher in patients with flat back than in patients with concave lumbar spine, with an odds ratio of 2.421 (0/557-1/163) (*P*: 0.227). The deformity of the vertebral column was not significantly correlated with the severity of SA (*P*: 0.256). The probability of SA was lower in the absence of deformity than having lumbar lordosis (OR: 0.663, *P*: 0.532). The probability of SA was lower in scoliosis than lumbar lordosis, (OR: 0.937, *P*: 0.97). The probability of SA was lower in the kyphosis than lumbar lordosis (OR: 0.613 CI: 0.044-0.094) (*P*: 0.650). Anatomical signs and symptoms had a significant relationship with the severity of SA (*P*: 0.001). The probability of a spinal anesthetized problem was lower in patients with visible lesions than in patients who were neither visible nor palpable to the caustic lesions, in that the odds ratio was 0.092 (*P*: 0.001). The probability of SA was lower in patients with unobservable and inaccurate strokes than patients who were neither visible nor visible to the caustic lesions, (OR: 0.283, *P*: 0.008). The distance between the vertebrae as a sequential variable with SA was statistically significant (*P*: 0.005), with a correlation coefficient was 0.337. The probability of SA was lower in patients with an easy intercostal distance than those with no inter-vertebral interval (OR: 0.088, *P*: 0.001). The probability of SA was a problem in patients who had difficulty touching the interval between the vertebras and those with no inter-vertebral intervals, (OR: 0.430, *P*: 0.144).

## DISCUSSION

Results of this study showed that age, weight, BMI of patients and the reduction of the ability to bend the waist and intervertebral space the not being touched and position of spinous processes can cause difficulty in performing spinal anesthesia in patients undergoing cesarean section. In a study by Atallah et al. performed on 300 patients with spinal anesthetic urology, bone anatomical markers and radiological features were indicated as two major factors in the prediction of difficult spinal anesthesia. In their study, BMI was expressed as an independent predictor for the difficulty of the spinal blockade.^9^

The findings regarding age, weight, and BMI showed that these factors are associated with an increased probability of difficulty in SA. In the cohort study performed by Stendell et al. on 73579 patients, BMI> 35 was expressed associated with difficulty in SA more than the other variables. Also, all factors such as age, BMI, history of difficult spinal anesthesia, anesthesia degree, surgical status in an emergency or outpatient setting, and difficult intubation were associated with difficult SA independently.^16^ Tessler et al., described the condition of the patient as an important factor for the success of the SA, which is affected by pregnancy, deformity of spine and old age. Similarly, the old age was described as a weak predictor factor in difficult SA.^17^

The results of the study showed that the quality of the touch of anatomical signs and the distance between the vertebrae were related to the difficulty of SA and the general appearance of the body was a poor indicator of prediction of difficult SA. Sprung et al., showed that there was no significant correlation between the factors like age, sex, type of needle, needle gauge and anesthetist’s experience and difficult SA. The appearance of the body regard to SA by definition of failure in the first attempt was not an effective factor in the prediction of difficult SA.^6^

In this study, there was a significant correlation between age, weight, BMI, touch quality of anatomical indices and difficult SA. However, height was not an effective factor in predicting difficult SA. The study by Kim et al., performed on 253 patients, also showed that the experience of the anesthesiologist, the distance between skin, the sub-arachnoid space, BMI and anatomical indicators were effective factors in the prediction of spinal anesthesia.^18^

We also showed that body appearance, duration of SA, the ability to bend the waist and touch quality of anatomical indices had a positive correlation with SA, but deformity of the vertebral column, height and the history of SA had no effect on prediction of SA. Chien et al., in a study on 848 patients, stated that anatomical indices had a strong correlation with SA. But the vertebral column deformity and appearance of the body were associated with difficulty of spinal anesthesia, considering the definition of increasing numbers of punctures, and the thoracic punctures were more predictive compared to the lumbar punctures.^7^ In the study of Filho et al. on 1481 patients, the anatomical indices of the spinal column, the anesthesiologist’s experience and patient’s status during SA were described as effective factors in determining the ease of SA.^19^ Our results showed that height, job and deformity of the vertebral column had no effect on the prediction of spinal anesthesia and the difficulty of performing SA.

### Limitations of the study

This is a single center study with almost appropriate sample size but for generalizability of results we need larger studies in diffierent situations, so theses results should be interpreted with caution.

## CONCLUSION

Results of this study showed that age, weight, BMI and the reduction of the ability to bend the waist, intervertebral space that not being touched and position of spinous processes can cause difficulty in performing SA in patients undergoing cesarean section. The results of this study can contribute to determining and designing a spinal blockade scoring system based on the patient’s characteristics and effective factors before the surgery, to facilitate the technique by anesthesiologist.

### Author’s contribution:

**SAKH:** Design of study, literature review, Review and final approval of manuscript.

**SS:** Analysis and manuscript writing. Review and final approval of manuscript.

**NA:** Helped in statistical analysis and literature review, Review and final approval of manuscript.

**NKh:** Data collection and analysis, Review and final approval of manuscript.

**MM:** Data collection and analysis, Review and final approval of manuscript.
